# Examining the Longitudinal Impact of Within- and Between-Day Fluctuations in Food Parenting Practices on Child Dietary Intake: Protocol for a Longitudinal Cohort Study Within a Sample of Preschooler-Parent Dyads

**DOI:** 10.2196/73276

**Published:** 2025-05-16

**Authors:** Katie A Loth, Julian Wolfson, Martha Barnard, Natalie Hogan, T James Brandt, Jayne A Fulkerson, Jennifer O Fisher

**Affiliations:** 1 Department of Family Medicine and Community Health Medical School University of Minnesota Minneapolis, MN United States; 2 Division of Biostatistics and Health Data Science School of Public Health University of Minnesota Minneapolis, MN United States; 3 School of Nursing University of Minnesota Minneapolis, MN United States; 4 Department of Social and Behavioral Sciences College of Public Health Temple University Philadelphia, PA United States

**Keywords:** food parenting practices, dietary intake, preschoolers, nutrition, eating behaviors

## Abstract

**Background:**

A healthful diet in early childhood is essential for healthy growth and disease prevention. Parents influence children’s diets through supportive (eg, structure and autonomy support) and unsupportive (eg, coercive control and indulgence) food parenting practices. Historically, much of this work has focused on parents’ “usual” feeding behaviors using survey methods. However, recent studies using ecological momentary assessment methods have allowed assessment of food parenting behaviors in “real time.” This work has revealed that the practices used by parents to feed children vary across contexts and are influenced by factors such as stress or time constraints. Research is needed to understand the dynamic nature of food parenting and its impact on children’s diets.

**Objective:**

This study aimed to describe the methods and procedures used in the Preschool Plates cohort study, which aimed to (1) describe within- and between-day fluctuations in food parenting practices across time and context, (2) examine the longitudinal impact of within- and between-day fluctuations in food parenting practices on child dietary intake, and (3) identify momentary predictors of within- and between-day fluctuations in food parenting practices across time and context.

**Methods:**

Preschool Plates is a longitudinal cohort study examining the impact of food parenting practices on the dietary intake of 3- to 5-year-old children. A total of 273 parent-preschooler dyads consented and enrolled, and 254 (93%) dyads completed baseline data collection. Dyads will be followed for 2 years using state-of-the-art measures, including an 8-day ecological momentary assessment protocol to assess food parenting, contemporary measures of food parenting, and 3 interview-led 24-hour dietary recalls, collected at baseline, 6 months, 12 months, and 24 months. Child height and weight will be measured at 3 time points.

**Results:**

Recruitment for our baseline sample (N=254) occurred between October 2023 and September 2024. Participants will complete follow-up data collection after 6 months, 12 months, and 24 months. A racially and ethnically diverse cohort was enrolled, with 28.3% (72/254) of enrolled participants identifying as White and 71.7% (182/254) identifying as non-White.

**Conclusions:**

Findings from the proposed study will inform the development of anticipatory guidance for feeding young children and randomized controlled trials designed to intervene on parents’ responses to momentary factors to encourage interactions with children around feeding that promote optimal diet quality. For example, findings could inform the development of an ecological momentary (ie, real time) intervention that delivers content to participants’ mobile devices in response to real-time assessments of context and circumstance.

**International Registered Report Identifier (IRRID):**

DERR1-10.2196/73276

## Introduction

### Background

A healthful diet during early childhood is important for healthy growth and development and helps protect against chronic diseases later in life [1]. Poor diet is a major contributor to chronic diseases, including diabetes, heart disease, stroke, and cancers, accounting for substantial morbidity and mortality [1]. In the last 2 decades, significant public health efforts have been made to improve children’s dietary intake [2-4], resulting in only low to moderate improvements in youth diet quality. Currently, more than half of youth consume poor quality diets, failing to meet dietary intake recommendations of the American Heart Association [4-6]. Importantly, significant disparities in diet quality exist across population subgroups, with youth from families with lower household income and food insecurity reporting persistently worse diet quality [4]. Disparities in diet quality during childhood contribute directly to disparities in morbidity and mortality associated with chronic disease across the lifespan [1,4].

Parents are key agents of influence in the dietary intake of their young children. Children’s eating behaviors and dietary intake are shaped significantly by their family and home food environment [7-13]. Parents influence their children’s eating through their use of food parenting practices [7-9,13]. Food parenting practices include a broad range of goal-directed actions and behaviors that involve what foods are made available and accessible, as well as how parents interact with their children around food [7]. The leading conceptual framework of food parenting, developed by Vaughn et al [7], describes 3 higher-level domains of practices: *structure*, such as food availability, accessibility, and limit setting; *autonomy support,* such as praise and reasoning; and *coercive control*, such as pressure to eat and overt food restriction. *Indulgence* has been proposed to be either a subdomain of structure [7] or a fourth unique high-level domain of potential importance [14,15]; indulgent behaviors allow children greater freedom over what, when, and how much to eat. Current theory and research to date suggest that food parenting practices within the structure and autonomy support domains are “supportive” of healthy eating in children, while the practices within the coercive control and indulgent domains are “unsupportive” [7-9,13]. However, empirical evidence to support the impact of structure and autonomy support practices on child outcomes is much more limited than the evidence base examining the short- and long-term impacts of coercive control practices [7,9].

Understanding momentary impacts on the use of specific food parenting practices is crucial to the future development of interventions aimed at shifting parents’ approach to feeding. Although experts generally agree that food parenting practices are goal-directed behaviors sensitive to circumstance, previous studies have typically assessed parents’ “usual” use of food parenting via questionnaires. Consequently, scientific understanding of food parenting is based on relatively gross indicators of the dynamic interactions around child feeding that fail to account for potentially important variation across time and contexts [7,16-19]. For example, a parent might report via survey that their “usual” use of coercive feeding practices is low, but when their stress level is high or their child’s behavior is challenging, they might pressure children to eat particular foods or place greater restrictions on children’s eating.

More recently, researchers have begun using ecological momentary assessment (EMA) to capture food parenting practices, as they unfold in their natural environment across time and context [15,20,21]. Findings from studies by Loth et al [15,20] revealed that most parents used a variety of theoretically supportive and unsupportive food parenting practices on a given day. Furthermore, in qualitative individual interviews, parents of preschoolers described the following momentary factors that influenced their use of specific food parenting practices: parent mood or stress level, child mood, behavior or physical health, time constraints, lack of planning, and competing priorities [14]. Parents in this study described shifting their feeding strategies in response to momentary factors, highlighting the need for more nuanced approaches to investigating within- and between-day variation in food parenting practices [15]. Recent evidence on temporal relationships between such momentary variables (eg, stress and child behavior) and use of food parenting practices supports this perspective [21,22]. This preliminary work demonstrated that momentary influences can shift parents’ engagement to food parenting practices that are unsupportive and associated with a higher risk of poor dietary intake over time. Developing an understanding of momentary factors that influence a parent’s use of particular food parenting practices across multiple contexts will allow for the development of effective interventions that address the important contexts and momentary factors that influence how parents interact with young children at meals and snacks.

Food parenting practices that support healthy eating in young children have not been fully established. Several gaps exist in the literature on food parenting practices [7,13,19]. First, the bulk of research exploring the impact of food parenting practices on diet-related outcomes has focused on coercive parenting practices [7,8,17]. Research focused on a broad range of food parenting practices is needed, given the broad scope of practices regularly used by parents. For example, many structure and autonomy support food parenting practices have received little (eg, guided choices [7]) or no (eg, food preparation [7]) attention in prospective studies. Second, the use and impact of distinct food parenting practices are often explored in isolation, although parents are more likely to use combinations of food parenting practices (eg, rules or limits and food restriction) [14,15,23].

Exploring food parenting practices across all 4 higher-order domains (ie, structure, autonomy support, coercive control, and indulgence), as well as various combinations of practices used, is necessary to tease apart overlapping constructs and behaviors. This approach provides a more nuanced, yet comprehensive, understanding of the impact of food parenting practices on children’s eating behaviors and dietary intake than is available in the current literature. Third, the bulk of studies on food parenting practices to date have been cross-sectional [7], making it difficult to draw conclusions about the temporal direction of the associations between food parenting practices and child outcomes, including dietary intake. Longitudinal studies with multiple data collection time points are required to ascertain the long-term impact of various food parenting practices on child dietary intake. Finally, until recently, most studies on food parenting practices have focused on samples that are predominantly White and higher income [7-9,16,17]. Preliminary research conducted within samples with more diversity suggests that families may differ, particularly with regard to race, ethnicity, parental education, or socioeconomic differences, in both the extent to which parents adopt a controlling approach to child feeding and the role that level of control within feeding strategies plays in child weight–related outcomes [22,24-26]. Enrollment of a cohort of parent-child dyads from racially, ethnically, and socioeconomically diverse backgrounds will allow for the exploration of associations of food parenting with child outcomes that are grounded in the cultural contexts of historically underrepresented groups.

### Objectives

The objective of this protocol paper is to provide a detailed description of the Preschool Plates cohort study methods. Preschool Plates is a population-based, longitudinal cohort study that was designed to improve upon the rigor of prior studies to advance our understanding of the use and impact of food parenting practices in important ways. Specifically, the Preschool Plates cohort study aims to: (1) describe within- and between-day fluctuations in food parenting practices across time and context, (2) examine the longitudinal impact of within- and between-day fluctuations in food parenting practices on child dietary intake, and (3) identify momentary predictors of within- and between-day fluctuations in food parenting practices across time and context.

Findings from Preschool Plates will deepen our understanding of how clinicians and public health practitioners can best support parents in adopting and maintaining the use of supportive food parenting practices despite momentary day-to-day challenges.

## Methods

### Study Design and Participants

Preschool Plates is a longitudinal cohort study examining the impact of food parenting practices on the dietary intake of children aged 3 to 5 years. At baseline, a total of 273 parent-preschooler dyads were consented and enrolled, and 254 (93%) dyads completed baseline data collection.

### Ethics Approval

Ethics approval was provided by the University of Minnesota Institutional Review Board (00018340). All caregivers provided informed consent, via an e-consent process, before the start of data collection. Due to the children’s age (<7 years), assent was not obtained. Once collected, study data are stored in Box Secure Storage, which is a system that is authorized for the use of protected health information. Data included in this protocol paper has been deidentified. Participants were provided with a financial incentive as a way of thanking them for their time and engagement; details on the specific compensation are included below under the subheading Incentives.

### Recruitment

Potentially eligible parent-child dyads were identified via MHealth Fairview primary care clinics. MHealth Fairview research services staff used electronic health records to identify current patients that were aged between 3 and 5 years and had been seen in a Fairview clinic within the previous 12 months. Only the parents of patients who had previously consented to being contacted for research studies were mailed a recruitment letter. This letter provided a brief description of the study and included a QR code where participants could provide their contact information and consent to being contacted by the Preschool Plates cohort study team.

A rolling recruitment approach was used, with letters sent to potentially eligible participants in batches until recruitment goals were met. Recruitment for our baseline sample occurred between October 2023 and September 2024. Initially, batches of potentially eligible participants were randomly selected from all potentially eligible participants. As recruitment progressed, targeted recruitment strategies were used with the goal of recruiting a racially and ethnically diverse sample. For a period of time, we intentionally mailed recruitment letters to patients from clinics located in more racially diverse neighborhoods, and at times, we limited mailings to individuals who identified as non-White. For detailed information on the demographics of the baseline sample enrolled, please refer to the Results section of this paper.

After potentially eligible participants indicated an interest in the study and provided consent to be contacted, a member of the Preschool Plates cohort study team reached out to them to complete a full eligibility screening. The inclusion criteria were as follows: (1) the target child aged is aged 3 to 5 years at baseline and lives in the same house as the reporting caregiver for ≥50% of the time, (2) the caregiver is primarily responsible for the child’s feeding outside of childcare, (3) the caregiver is able to read and speak English or Spanish, and (4) the child has visited an MHealth Fairview clinic in the previous 12 months. The exclusion criteria were as follows: (1) caregiver is aged <18 years; (2) child has a history of major food allergies (eg, peanuts); (3) child uses medication (eg, insulin), has a developmental disability (eg, autism), or has a medical condition (eg, diabetes) that affects food intake and growth; and (4) child is in foster care.

**Figure 1:**
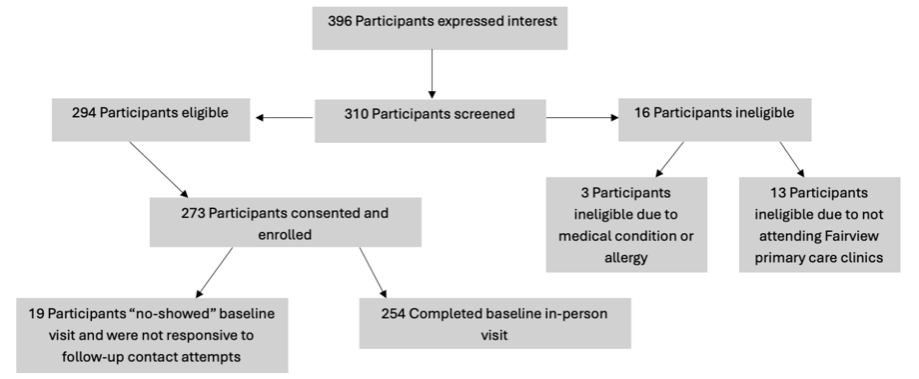
CONSORT (Consolidated Standards of Reporting Trials) flowchart for the Preschool Plates cohort study.

Participants who were determined to be eligible and who remained interested in participating were engaged in the informed consent process over the telephone and scheduled for their baseline visit. Following this telephone conversation, an e-consent form was signed by participants. Figure 1 is a CONSORT (Consolidated Standards of Reporting Trials) diagram that outlines the recruitment, consent, and enrollment process for baseline data collection.

### Data Collection Procedures

#### Overview

During their initial enrollment call, participants were briefed on data collection procedures, engaged in the informed consent process, and were scheduled for the in-person baseline visit.

At the baseline visit, participants received additional details on measurements and the data collection schedule and were trained on protocols for surveys, EMA, and 24-hour dietary recalls. Parents completed the baseline survey at this visit. Finally, height and weight measurements for the child participant were completed at this time. In total, Preschool Plates includes 4 data collection sessions over 2 years: baseline (T1), 6 months (T2), 12 months (T3), and 24 months (T4). T1, T3, and T4 include in-person data collection at the University of Minnesota; T2 includes only remote data collection activities.

At each time point, participants completed a survey to assess usual use of food parenting practices as well as additional parental, household, and environmental factors of interest. They also completed three 24-hour dietary recalls, reporting daily dietary intake on behalf of their child, to assess overall quality of diet and participated in 8 days of EMAs. Children’s height and weight were measured by trained study staff at T1, T3, and T4.

All participant-facing study materials were available in English and Spanish. Materials were professionally translated and then further reviewed by bilingual study staff. Bilingual study staff were also employed to engage with participants who were Spanish speaking. Data collection methods and measures for study variables of interest are presented below and summarized in Table 1.

**Table 1:** Constructs of interest, study measures, and methods.

Measurement methods and constructs				Description of measure
**EMA^a^**				
	Signal prompts: salient momentary factors			EMA measures of salient momentary factors [14,20,21,27,28]
	Event prompts: food parenting practices			Updated version of the Real-Time Parent Feeding Measurement Tool [15] and Child Dietary Intake [29,30]
24-hour dietary recalls: dietary intake				HEI^b^ total, component scores: HEI-2015 [31,32] and HEI-2022 [32]
Surveys: food parenting practices				Comprehensive Feeding Practices Questionnaire [33], Food Parenting Inventory [34], Structure and Control in Parent Feeding [35], and Parenting around Snacking Questionnaire [36]
**Covariates**				
	**Child-level factors**			
		Child developmental stage	Ages and Stages Questionnaire [37]	
		Child temperament	Children’s Behavior Questionnaire-Short Form [38]	
		Mood	Positive and Negative Affect Scale for Children [27,39]	
		Behavior	Strengths and Difficulties Questionnaire [40,41]	
		Time spent in day care	Days, hours, and a typical schedule	
	**Parent-level factors**			
		Parenting style	Parenting Practices Questionnaire [42]	
		Global and mental health	NIH PROMIS^c^ measures [43]	
	**Family-level factors**			
		Household food security	USDA^d^ Food Insecurity 18-item long form [44,45]	
		Acculturation	Acculturation Rating Scale for Mexican Americans [46]	
		Household structure	Survey measures to be developed	
	Demographics (for example, race or ethnicity, and income)			Survey measures to be developed
	In-person measurements: height and weight			BMI and weight status

^a^EMA: ecological momentary assessment.

^b^HEI: Healthy Eating Index.

^c^NIH PROMIS: National Institutes of Health Patient-Reported Outcome Measurement Information System.

^d^USDA: United States Department of Agriculture.

#### Ecological Momentary Assessment

EMAs were used to assess specific food parenting practices used during shared eating occasions, as well as to identify momentary predictors of within- and between-day variability in use of specific food parenting practices.

The EMA protocol included 2 distinct types of assessments. the first was event-contingent assessments; for these, caregivers were asked to complete an EMA survey after every eating occasion during which they were present with the enrolled child. Event-contingent assessments were initiated by caregivers after each shared eating occasion (eg, meals or snacks); caregivers simply used their phone to click on a study-provided link. Event-contingent assessments included questions on food parenting practices and the child’s food intake during that specific eating occasion. On average, participants took 4.41 (SD 6.35) minutes and a median of 2.77 minutes (IQR 1.90-4.32) to complete each event prompt. The second was signal-contingent assessments, for which caregivers were texted to complete a survey at random intervals situated within 4 time blocks throughout the day (eg, 7 AM-10 AM) to capture momentary variables of interest. Signal-contingent prompts were timed to expire after 1 hour of receipt of the text prompt. On average, participants took 1.74 (SD 2.71) minutes and a median of 1.22 (IQR 0.85-1.82) minutes to complete each signal prompt.

Caregivers were asked to complete 8 days of EMA data collection. A day was considered to be complete if they engaged in 2 event-contingent and 2 signal-contingent assessments; if a parent failed to complete adequate prompts on a specific day, they were given an additional day to complete the required prompts. At baseline, on average, it took parents 9.667 (SD 3.63 days; median 9.0, IQR 8-10) days to achieve 8 complete days. To minimize potential confounding effects of weekly rhythms (eg, weekday vs weekend variations in behavior, mood, or context), all participants began data collection on the same day of the week. This approach, which followed recommendations in the extant EMA literature [47-50], ensures that each participant’s data collection period spanned identical calendar days.

Food parenting practices were assessed using an updated version of the EMA-based Real-Time Parent Feeding Practices survey tool [15]. This tool was developed by Loth et al [15] for the Kids EAT! study (described below in the Pilot Study section) to measure the real-time use of a broad range of food parenting practices within an EMA protocol and was updated with additional items for the Preschool Plates cohort study. The modified version of the Real-Time Parent Feeding Practices Assessment tool included 36 questions on food parenting practices situated within 4 higher-level theoretical domains, including structure (14 items), autonomy support (8 items), coercive control (6 items), and indulgent (8 items). Individual questions were designed to measure specific subconstructs, as outlined in the content map of fundamental constructs in food parenting practices by Vaughn et al [7], drawing from existing questionnaires where possible, such as the Child Feeding Questionnaire [18] and the Food Parenting Inventory [34]. These questions were adapted for use within an EMA protocol.

For example, an item on the Child Feeding Questionnaire was designed to measure parental pressure to eat and read, “I have to be especially careful to make sure my child eats enough.” This question was adapted for this study to focus on a parent’s specific behavior at the most recent meal or snack consumed by their child. The adapted question read, “At this meal or snack, did you have to encourage [your child] to eat more food than they wanted to?” (subconstruct: pressure to eat; higher-order domain: coercive control).

Additional example items within the Real-Time Parent Feeding Practices survey tool included the following:

- “At this meal or snack did you give [child] the choice of several specific options and let the child pick?” (subconstruct: guided choices; higher-order domain: structure)
- “Did you involve your child in helping to plan, purchase or prepare the foods that were served?” (subconstruct: child involvement; higher-order domain: autonomy support)
- “At this meal or snack did you try to avoid conflict by only preparing/serving foods that you knew your child likes/will eat?” (subconstruct: anticipatory catering; higher-order domain: indulgence).

Parents were asked to report on their use of all 36 food parenting practices following each shared eating occasion.

Eating occasion–level child dietary intake was assessed via a measure developed for and validated against the interview-led 24-hour diet recall for use within an EMA protocol [29,30,51]. Parents were asked which categories of foods were eaten by the target child at the eating occasion (yes or no): fruit; vegetables; whole grains (eg, whole grain breads or cereals, brown rice, oatmeal, and corn tortillas); refined grains (eg, white bread or cereals, flour tortillas, white rice, and crackers); dairy (eg, milk, cheese, yogurt, milk alternate such as soy milk, and ice cream); meat protein (eg, chicken, beef, and seafood or fish); beans, eggs, seeds, nuts, and tofu; sugary drinks (eg, pop, Kool-Aid, Capri Sun, Sunny Delight, and sports drinks); cake, cupcakes, cookies, or other baked goods; and candy (eg, sweets, chocolate, Gushers, and fruit snacks). When asked about food served at the meal, participants were also given the following guidance, “For a dish containing multiple foods (e.g. soups, sandwiches and casseroles), please select the main ingredients in the dish.” For example, if you had beef and vegetable soup, mark both “meat protein” and “vegetables.”

From these responses, 10 individual food categories were created, including fruit, vegetable, whole grain, refined grain, dairy, meat protein, plant protein, sugary drinks, baked goods, and candy. A modified version of the Healthfulness of the Meal Index [29], which was adapted from the Healthy Eating Index (HEI) categories [52], was also calculated. In alignment with prior research, the Healthfulness of the Meal Index was scored using an eaten or not eaten format. For example, an eating occasion received 1 point for the child consuming each of the following foods (fruit, vegetable, whole grain, dairy, meat protein, and plant protein) and 1 point for not eating each of the following foods (refined grains, sugary drinks, baked goods, and candy). Points were then summed with a range of 0 to 10 for each eating occasion. A similar Healthfulness of the Meal Index has been used within studies using EMA protocols to conduct research within similarly diverse populations of children [53]. This measure of eating occasion–level dietary quality provided direct correspondence between food parenting practices used at each eating occasion and types of foods eaten or overall healthfulness of the foods eaten, at the same, or subsequent eating occasions, allowing for an understanding of within-meal, and within-day associations between food parenting practices and dietary quality (refer to hypothesis 3 below in the Results Section).

Momentary predictors were assessed using a combination of items developed and pilot-tested as a part of the Kids EAT! study [15,20], as well as items developed or modified for use within an EMA protocol by others. Parents reported on their current mood [27] and stress level [22], as well as their child’s current mood [28], using tools validated for use within a similar population and within an EMA protocol. Finally, additional momentary factors were assessed by asking parents to report on their experience of 12 specific factors identified within previous qualitative research as salient predictors of food parenting practices [14]. For example, parents responded to the following statement regarding how they had felt since the last assessment: “Busy with a number of work or household activities” (responses were evaluated on a 5-point Likert scale, ranging from 1=very slightly or not at all to 5=extremely).

#### Surveys

The baseline survey was designed to assess usual use of food parenting practices as well as additional parental, household, and environmental factors of interest. Participants completed the same survey at each time point (T1-T4). This survey was administered on the web via REDCap (Research Electronic Data Capture; Vanderbilt University), a secure, web-based data collection platform sponsored by the University of Minnesota and provided by the Clinical Translational Research Institute [39]. Participants took an average of 30 to 45 minutes to complete the survey. To enhance engagement, we incorporated visual elements within REDCap, including our Preschool Plates cohort study logo, motivational messages (eg, “Over Halfway Done! Keep it Up!”), and a progress bar to help participants track their completion status.

In the survey, food parenting practices were assessed using subscales from the following 4 questionnaires: Comprehensive Feeding Practices Questionnaire [33], Food Parenting Inventory [34], Structure and Control in Parent Feeding [35], and Parenting around Snacking Questionnaire [36]. In addition, covariates and effect modifiers of interest at the child level (eg, developmental stage [37], temperament [38], mood [39], and hours spent in day care), parent level (eg, parenting style [42] and mental health [43]), family level (eg, acculturation [54] and food insecurity [44,45]), child-level demographics (eg, age, race or ethnicity), parent-level demographics (eg, educational attainment and employment status), and family-level demographics (eg, public assistance, income, and socioeconomic status) were assessed via this survey.

#### 24-Hour Dietary Recalls

Interviewer-led 24-hour dietary recalls were used to assess the quality and quantity of child dietary intake. Three 24-hour dietary recalls were collected for each child participant at baseline (T1) and each follow-up period (T2-T4). General availability to complete recalls was gathered for each participant individually by data collectors during their in-person visit. Participants had the ability to provide their availability for every day of the week, including weekends. Participants were given an 8-day window to complete their recalls, with a hard stop 14 days after their start date. The date and time for recalls were random but based on participants’ provided availability. Recalls were completed by a team of trained and certified staff based out of the Nutrition Coordinating Center using the Data System for Research [55].

A multiple-pass interview technique was used to prompt for a complete food and beverage recall and descriptions. Because preschool-aged children were not considered reliable reporters of dietary intake, recalls were conducted with the index child’s enrolled parent using protocols developed and validated by the Nutrition Coordinating Center for the collection of data on the dietary intake of young children [52]. Parents were provided with multiple resources to assist with dietary intake reporting, including the Food Amounts Booklet (designed for use with young children) to help estimate portion sizes. To reduce missing data and minimize bias from parental assumptions about foods consumed in their absence, we also provided the Foods Fed by Other Adults handout, which could be completed by individuals such as day care providers when the enrolled parent was not present.

Intake for the 3 recalls was averaged across days to create mean scores for intake of food categories of interest (eg, fruits, vegetables, sugary beverages, and salty and sweet snacks). Furthermore, HEI-2022 [32] total and component (eg, added sugars and sodium) scores will be calculated at each data collection time point (T1-T4) using methods designed for estimating overall dietary quality for an individual within an etiologic study, specifically the multivariate Markov chain Monte Carlo approach [56,57]. HEI is a scoring system that was developed to measure the degree to which a diet is consistent with the Dietary Guidelines for Americans and is a valid and reliable measure of diet quality [58]. The closer an HEI score is to 100 (range 0-100), the more aligned it is with the Dietary Guidelines for Americans [58,59]. The HEI-2022 total score is the sum of 13 subcomponent scores that measure adequacy (total fruits, whole fruits, total vegetables, greens and beans, whole grains, dairy, total protein foods, seafood and plant proteins, and fatty acids) and moderation (refined grains, sodium, added sugars, and saturated fats) [32]. This measure of overall dietary quality will help us to understand the longitudinal impact of specific patterns of food parenting on the child’s overall diet quality over time (at 6-month intervals for 2 years; refer to hypothesis 4 in the Results section).

#### Anthropometric Measurements

At the T1, T3, and T4 data collection visits, child participants’ height and weight were measured by trained study staff following a standardized protocol. For each visit, children were measured using a calibrated digital scale (to the nearest 0.1 kg) and a stadiometer (to the nearest 0.1 cm). Two measurements of height and weight were taken, and if the values differed by >0.5 units, a third measurement was collected. The final recorded values were determined by averaging the 2 closest measurements. All data collectors underwent training on proper measurement techniques, including instructions on positioning the child, ensuring minimal clothing, and verifying equipment calibration [53,54]. Data collectors underwent retraining every quarter. A measurement log was maintained within REDCap to record each measurement and any procedural notes.

In the event a family could not attend the on-campus visit for anthropometric measurements, parent consent was obtained to access the child’s medical records from the MHealth Fairview health system. Children whose data were extracted from electronic health records were required to have seen a health care practitioner within the preceding 6 months.

### Retention Efforts

Retention strategies focused on consistent communication to ensure participant engagement and adherence. Participants received regular reminders to complete EMA, dietary recalls, and surveys. Study staff and data collectors were trained to follow up with participants promptly, typically within 1 business day, to address any questions, delays, or noncompliance.

Participants scheduled for in-person visits were contacted via text or phone calls to confirm the date, time, and location. Confirmation emails provided detailed logistical information, including GPS coordinates, parking instructions with photos, and other relevant details. Reminder texts were sent the day before visits to reinforce appointment schedules and logistical information.

Postcards were sent at multiple time points to maintain participant engagement and provide reminders for study activities. Mother’s Day and Father’s Day postcards were sent to all enrolled participants. Annual birthday postcards were mailed to parents and children during the study period. In addition, participants were sent a postcard reminder 2 weeks before remote data collection windows (T2) and 1 week before in-person visits (T3 and T4).

Participants returning for in-person visits at T3 received personalized coloring books created by study staff. This coloring book was tailored to the study’s preschool-age population and was designed to encourage participant involvement in follow-up visits.

### Incentives

Participants were compensated for their time and effort across the 5 data collection periods, with a maximum potential compensation of US $950 over the 2-year study period. At the baseline visit (T1), participants were provided with a Greenphire ClinCard, a reloadable payment card, which was used to distribute incentives throughout the study. Compensation was structured to reflect the time and effort required for specific study components. Surveys were completed at each time point (T1-T4) and were incentivized with US $50. For each set of three 24-hour dietary recalls, participants received US $25 per recall, totaling US $75 per data collection point. Completion of the 8-day EMA protocol was compensated with US $75 per time point. For anthropometric measurements collected in person at T1, T3, and T4, participants received US $50 per visit. If anthropometric data were collected from medical records rather than in person, no payment was provided for that time point’s measurement. This incentive structure was designed to equitably compensate participants while encouraging engagement and retention throughout the study.

### Pilot Study

Kids EAT! was a National Institutes of Health–funded K23 study (principal investigator: KAL), which used mixed methods (interviews, surveys, and EMA) to explore the real-time use of food parenting practices within a racially, ethnically, and socioeconomically diverse sample of parents of preschool-aged children (n=120) [15,20,60,61]. EMA protocols for Kids EAT! included asking parents to respond to a series of signal- and event-contingent prompts delivered to their smartphones. Results from Kids EAT! demonstrated feasibility in using EMA with parents from diverse backgrounds and collecting data from parents working shift work, during time periods when children were in school or day care, and from parents with differing levels of comfort with technology. The Preschool Plates cohort study protocol used the successful staff training models, data collection protocols, and participant incentive plans used in the Kids EAT! study. Kids EAT! parent participants were successfully engaged in several different data collection approaches similar to those proposed in this study, including web-based surveys and EMA.

### Statistical Analysis Plan

#### Overview

R (R Foundation for Statistical Computing) will be used for data cleaning and main analyses. Distributions of key outcome variables will be assessed and transformed if necessary. Unless otherwise specified, hypothesis tests will be conducted using a 2-sided α level of .05.

EMA responses will be aggregated to derive scores for each of the 4 food parenting practice domains during each data collection period. Given the unequal number of questions related to each domain, 2 versions of domain scores will be created by computing (1) the number of items endorsed in each domain and (2) the proportion of items endorsed in each domain. Domain scores will be calculated for responses given at each event prompt (typically 2-8 per day over each 8-day data collection period).

Food parenting practice domain scores will be aggregated and compared visually and statistically at the event level (eg, breakfast, lunch, snack, and dinner) and by time period (eg, 9 AM to noon, 3 PM-5 PM, and 5 PM-9 PM). In addition, for each data collection period, we will fit linear mixed models for each domain of the form (M1) *D_ij_ = β_0_ + β_1_Z_ij_ + β_2_X_i_ + b_i_ + ε_ij_*, where *D_ij_* is the domain score for person *i* at event prompt *j*, *Z_ij_* is the event characteristic of interest (meal or time period indicator), *X_i_* is a set of baseline characteristics for person *i*, *b_i_* is a person-specific random effect, and *ε_ij_* is the residual. Longitudinal response patterns in domain scores will be visualized and qualitatively compared. We will calculate each individual’s SD, IQR, and entropy of domain scores across their longitudinal observations. How between-day fluctuations in food parenting differ across individuals will be quantified using longitudinal mixed models, similar to those described above, with the event characteristics *Z_ij_* representing between-day factors such as day of the week. The SD, IQR, and entropy of residuals from these models will also be summarized to characterize between-day fluctuations.

The distribution of the summary measures (SD, IQR, and entropy) of the longitudinal models described above will be used to derive measures of the consistency and endurance of food parenting practices. A metric of within-day consistency across event characteristics (eg, meal type) will be derived by calculating the distribution of SDs of individual residuals from model (M1) and then calculating an individual’s *z* score relative to this distribution. For example, an individual whose food parenting practices fluctuate substantially across meals will have a large SD of residuals relative to the population, so their *z* score will be relatively large. Consistency *z* scores will be used as predictors of child dietary intake in aim 2. Endurance will be measured by applying a similar *z* score approach but with models that incorporate data across multiple data collection periods. For example, model (M1) will be expanded to include data from times T2 to T4, and endurance *z* scores will be calculated from the distribution of spread summary measures (SD, IQR, and entropy) of the residuals from this expanded model.

Individual food parenting practice domain scores, as well as consistency and endurance metrics, will be used as inputs to a k-means algorithm (implemented using the *kmeans* package in R) to identify clusters of parents observed to use similar food parenting practices over the data collection period. For example, this technique might distinguish subsets of individuals who consistently and primarily use structure and autonomy support practices and those who use these practices less consistently or more commonly use coercive and indulgent practices. We will apply the k-means algorithm with a modest number of clusters (3-5) to ensure that the resulting groups are of sufficient size to accurately characterize them. Descriptive statistics will be used to summarize and compare the food parenting practices as well as demographic and family characteristics of each cluster. Cluster stability metrics and qualitative assessment of the scientific meaningfulness of the clusters will be used to choose a final number of clusters that will define subgroups for inclusion in the following analyses.

To examine the longitudinal impact of within- and between-day fluctuations in food parenting practices on child dietary intake, we will fit regression models for the outcome of child HEI measured from 24-hour dietary recall at time points T2 to T4. The main predictors of interest will be the individual-level summary measures of the 4 food parenting practice domains derived above. Our primary models will include the subgroup indicators classifying parents according to food parenting practice patterns, but we will also consider models that use average domain scores as well as consistency and endurance metrics. We will fit separate linear regressions for each data collection period, as well as longitudinal models that incorporate data across the time points T2 to T4. For period-specific models, we will include cluster indicators as well as consistency *z* scores that reflect the degree of within- and between-day fluctuations in food parenting practices during that period, along with relevant adjustment covariates. For longitudinal models, we will also include endurance *z* scores that reflect whether food parenting practices are maintained across data collection periods.

To identify momentary predictors of within- and- between-day fluctuations in the use of food parenting practices across time and context, we will combine the signal and event EMA survey data to understand how momentary factors (eg, mood, stress, and time constraints) affect food parenting practices. The main statistical approach will be to fit longitudinal linear mixed models of the form: (M2) *D_ij_* = *β_0_* + *β_1_W_ij_* + *β_2_X_i_* + *b_i_* + *ε_ij_*. Here, *W_ij_* represents a *vector* of momentary predictors, which will be derived by summarizing signal surveys within fixed time windows before the event *j*. Initially, we will define 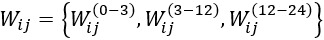, with the superscript in parentheses indicating the number of hours before the event covered by that variable. For instance, 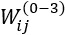 could reflect the average level of stress reported from signal surveys responded to in the 3 hours preceding the given event survey. We will fit separate models for each domain score during each data collection period; to increase power, we will also fit combined models that incorporate responses across multiple data collection periods. Single-predictor models (involving 1 type of momentary predictor, eg, stress) as well as multipredictor models will be considered. Time windows will be adjusted based on the observed response patterns of data, and if the momentary predictor data are overly sparse, multiple imputation will be used to fill in missing *W*values. In exploratory analyses, we will investigate whether momentary predictors interact with each other or with fixed and summarized individual characteristics (ie, consistency and endurance *z* scores) to impact food parenting practices. This will allow us to explore whether the strength of associations between momentary predictors and food parenting practices varies based on individuals’ levels of volatility in these practices.

Some participants may respond sporadically to event prompts, resulting in gaps between event prompt responses, which make it difficult to assess longitudinal patterns. We will begin by characterizing missing data patterns by participant and temporal factors, including the end-of-day prompts asking participants to indicate why they did not respond to prompts on a given day. Then, we will define and analyze a subset of “regularly responding” individuals for which there is sufficient data to characterize between-day fluctuations in food parenting practices, for example, a subset of individuals who have at most 2 days of event prompt nonresponse during their data collection period. To explore overall patterns and associations with non-EMA outcomes, we will augment this regularly responding set with data from individuals with more sporadic response patterns but who, based on their responses to end-of-day prompts, more plausibly have responses missing at random (eg, reported “forgot” rather than “too stressed”).

Food parenting practices and their associations with other measures may vary across subgroups defined by, for example, child developmental stage and parent cultural background. While our power to formally test for effect heterogeneity across these subgroups is limited, we will describe these differences by including interaction terms for these subgroups in the regression models described above.

#### Power Analysis

Given that the goal of this study is to describe patterns and associations and not evaluate a particular intervention, there is no formally defined primary end point. Hence, our power analysis focuses on describing the effect sizes that can plausibly be detected with the given analytic sample size of 200 families (accounting for approximately 20% attrition from our baseline sample).

Key outcomes are the mean levels of the 4 food parenting practice domains. With our sample size, 95% CIs for these means based on the total sample will have a margin of error of +0.17 or –0.17 SDs, corresponding to a small effect size per usual guidance [62]. For the clustering analyses, most participants will have at least 32 longitudinal observations spanning 8 days of data collection. Hence, we anticipate having an adequate sample size to accurately calculate individual summaries of the mean use, consistency, and endurance of food parenting practices, which will be used in the k-means clustering algorithm. To ensure that we can adequately characterize clusters based on demographic characteristics, we plan to restrict our clustering approaches so that they identify no more than 5 clusters, yielding an average cluster size of at least 30 participants; we will also restrict the minimum cluster size to 15 participants.

We calculate the detectable effect size of an analysis comparing mean child HEI between 2 groups of size n=100, for example, comparing families above and below the median of food parenting consistency or endurance. We have 80% power to detect a difference of 0.4 SDs, corresponding to the difference of approximately 4.7 units in total HEI given an HEI SD of 11.6 units in children aged 2 to 4 years enrolled in the NET-Works study [63]. For comparing 2 groups of size n=50 (eg, quartiles), we will have 80% power to detect a difference of 0.57 SD, or 6.7 HEI units. Power for associations between continuous summary measures of food parenting practices and total HEI will be higher.

The study will yield at least 32 observations per person (4 event or signal responses per day ×8 days), resulting in a total of 6400 data points on each domain. We conducted a simulation to assess the power to detect an association between a binary covariate and a domain score using parameter values informed by data from Kids EAT! [15,20]. For covariates that vary only between individuals, we estimate 80% power to detect a difference of 0.55 items endorsed per signal survey for a given domain. Because the maximum number of possible items endorsed per signal survey ranges from 4 to 7 across domains, an effect of this magnitude seems plausible. For momentary predictors, we have 80% power to detect differences of 0.14 items endorsed per signal survey. In general, power calculations using binary covariates tend to be more conservative than those using continuous covariates; therefore, we anticipate the actual power to be higher than estimated in this simulation.

## Results

### Overview

Recruitment and data collection began in October 2023. At the time of this protocol paper’s submission, data collection is still in progress and is expected to continue through November 2026. A racially and ethnically diverse cohort was successfully enrolled, with 28.3% (72/254) of enrolled participants identifying as White and 71.7% (182/254) identifying as Black, Indigenous, or people of color. Refer to Table 2 for detailed information on the characteristics of the Preschool Plates baseline sample.

Hypotheses

This study hypothesized the following:

1. Use of food parenting practices will vary within- and between-days; unique patterns will emerge across time (eg, time of day and day of week) and context (eg, location and type of eating occasion).
2. Distinct patterns in food parenting approaches will emerge; distinct pattern features will include mean levels of engagement in specific food parenting practices (by domain), as well as consistency in approach across day-, week-, and 6-month intervals, and endurance (or lack thereof) of patterns across time.
3. Healthful eating occasion–level dietary quality (ie, Healthfulness of Meal Index assessed via EMA) will be positively associated with supportive feeding practices (eg, structure and autonomy support) and inversely associated with unsupportive practices (eg, coercive control and indulgent).
4. Consistent and enduring use of supportive feeding practices will be associated with a more healthful overall diet quality (ie, HEI assessed via 24-hour dietary recalls) cross-sectionally and at 2-year follow-up, as compared to inconsistent or short-lived use of supportive practices and consistent and enduring use of unsupportive practices.
5. We expect to identify numerous momentary predictors of significance (eg, mood, stress, child behavior, and time constraints). Parents will shift within the day from supportive feeding practices toward unsupportive feeding practices in response to momentary predictors (eg, high stress, low mood, or limited time).

**Table 2:** Characteristics of the Preschool Plates baseline sample (N=254).

Characteristics		Values
Child age (y), mean (SD)		3.48 (0.59)
**Child’s sex, n (%)**		
	Male	144 (56.7)
	Female	108 (42.5)
	Unknown	2 (0.8)
**Child’s race or ethnicity, n (%)**		
	Asian	44 (17.3)
	Black	42 (16.5)
	Hispanic	15 (5.9)
	Mixed race	77 (30.3)
	Native American	2 (0.8)
	White	72 (28.3)
	Unknown	2 (0.8)
**Parent educational attainment, n (%)**		
	High school or less	26 (10.2)
	Technical college graduate or some college	43 (16.9)
	College graduate	78 (30.7)
	Graduate degree	107 (42.1)
**Parent employment status** **, n (%)**		
	Not currently employed	45 (17.7)
	Part time (<40 h/wk)	66 (26)
	Full time (>40 h/wk)	143 (56.3)
**Income (US $), n (%)**		
	<50,000	51 (20.1)
	50,000-74,999	42 (16.5)
	75,000-99,999	34 (13.4)
	>US $100,000	125 (49.2)
	Unknown	2 (0.8)
Participation in ≥1 forms of government assistance, n (%): yes		110 (43.3)

## Discussion

### Principal Findings

The overarching goal of the Preschool Plates cohort study is to advance the evidence base of food parenting approaches for preventing poor dietary intake among children. We argue that parents’ feeding practices vary across time and context and that a deeper understanding of variability and associated outcomes is necessary to inform the design of interventions to help parents consistently use supportive feeding practices despite challenging circumstances. Study findings will shed light on the developmental trajectory of the parent-child feeding relationship and reveal both the short- and long-term effects of food parenting practices on children’s dietary intake. Long-term findings will be used to inform the future development of interventions designed to be responsive to momentary factors found to interrupt parents’ use of supportive food parenting practices.

### Conclusions

To the best of our knowledge, the Preschool Plates cohort study is the most comprehensive study to date to examine the real-time use and the short- and long-term impact of food parenting practices on child dietary intake. Findings will guide the future development of a randomized controlled trial using ecological momentary interventions or a real-time intervention that delivers intervention content to participants’ mobile devices in response to real-time assessments of context, behavior, and circumstance, with the goal of improving children’s dietary intake and consequently reducing the morbidity and mortality associated with chronic disease across the lifespan.

## Abbreviations

CONSORT: Consolidated Standards of Reporting Trials
EMA: ecological momentary assessment
HEI: Healthy Eating Index
REDCap: Research Electronic Data Capture
